# The Mental Health Impact of the COVID-19 Pandemic on Healthcare Workers in the Eastern Mediterranean Region: A Scoping Review

**DOI:** 10.3389/ijph.2022.1604814

**Published:** 2023-01-04

**Authors:** Thaer Alhroob, Walaa Abu Alya, Beesan Nader Maraqa, Carmel Jaser Khalil, Aisha Shalash, Niveen M. E. Abu-Rmeileh, Zaher Nazzal

**Affiliations:** ^1^ Ministry of Health, Ramallah, Palestine; ^2^ Department of Family and Community Medicine, Faculty of Medicine, Hebron University, Hebron, Palestine; ^3^ Department of Medicine, Faculty of Medicine and Health Sciences, An-Najah National University, Nablus, Palestine; ^4^ Institute of Community and Public Health, Birzeit University, Birzeit, Palestine

**Keywords:** anxiety, burnout, depression, stress, insomnia, healthcare workers, East Mediterranean region, COVID-19

## Abstract

**Objectives:** This scoping review is to investigate the existing literature on the mental health of Healthcare workers, including stress or distress, anxiety, depression, burnout, insomnia, and fear or phobia within the different countries in the Eastern Mediterranean region (EMR) during the COVID-19 pandemic.

**Methods:** We systematically searched to consolidate studies across EMR countries regarding the mental health morbidity studied, the scales, and the methodology used. The review focused on peer-reviewed academic literature published from March 2020 to November 2021.

**Results:** One hundred sixty-seven articles were included in the review. Most publications came from lower-middle-income countries such as Iran, Pakistan, and Egypt. Most of the literature was specific to Stress/Distress (*n* = 94), followed by anxiety (*n* = 93), depression (*n* = 66), burnout (*n* = 27), insomnia (*n* = 20), and fear/phobia (*n* = 12).

**Conclusion:** Fear, phobia, and insomnia have all been examined extensively worldwide, yet they were among the Eastern Mediterranean region’s least explored outcomes. In addition, most underdeveloped countries have a low rate of publication.

## Introduction

In 2019, the World Health Organization (WHO) declared the novel coronavirus (COVID-19) outbreak a pandemic [1]. Healthcare workers (HCWs) are at high risk of acquiring this infection worldwide [2, 3]. HCWs are well-known to be under great stress on the job, and infectious illness epidemics exacerbate the situation [4, 5]. Apart from the physical risk of infection, the pandemic’s impact on the mental health of HCWs has escalated.

Under normal circumstances, one in four people worldwide is affected by mental health concerns [6]. HCWs are particularly vulnerable to experiencing mental illness; generally, they face a significant threat to their physical, psychological, and social wellbeing due to the nature of their work [7]. They are also expected to deal with patients’ painful experiences and fear of contracting certain diseases and passing them on to their families, as was the case during the COVID-19 pandemic [8]. Additionally, HCWs are susceptible to negative health consequences associated with poor sleep quality and feelings of isolation [9]. That is why it is essential to focus on this population mainly and learn more about their concerns. HCWs’ mental health is associated with improved wellbeing, patient safety, and quality of care.

Numerous mental health symptoms, including depressive symptoms, psychological distress, post-traumatic stress disorder, and resilience, have been studied concerning the impact of the COVID-19 pandemic on HCWs [10]. This was due to COVID-19 work-related duties, including a lack of protective equipment, an ongoing risk of contamination, worry about family members, and fear of death [11]. A meta-analysis revealed that symptoms of depression, anxiety, insomnia, post-traumatic stress disorder, phobia, obsessive-compulsive disorder, and somatization were highly prevalent during the COVID-19 pandemic. Therefore, it was recommended that HCWs who may be at risk of developing mental health difficulties should be given prompt and adequate screening and treatment [12]. Another systematic review revealed a considerable prevalence of depression, anxiety, insomnia, and stress among healthcare workers due to the pandemic [13].

The Eastern Mediterranean Region (EMR) consists of 22 countries with varying economic statuses and health challenges that extend from Pakistan in the east to Morocco in the west, with a population of nearly 645 million people [14]. Additionally, it has diverse healthcare systems; over the last decade, at least ten nations in the region have been or continue to be occupied, embroiled in internal war, or confronted with a challenging emergency [15].

In EMR, HCWs reported high rates of mental health issues when asked about COVID-19 [16]. However, the literature on the mental health consequences of HCWs working in the EMR during the COVID-19 pandemic is expanding, and no comprehensive review is available. Therefore, following a thorough review of the literature, we opted to include depression, anxiety, stress, insomnia, fear, and phobia in our study, as these were the most studied topics.

The main goal of this scoping review is to investigate the existing literature on the mental health of HCWs, including stress/distress, anxiety, depression, burnout, insomnia, and fear or phobia within the different countries in the EMR during the COVID-19 pandemic. In addition, we will also focus on the different types of mental health issues frequently studied and the various instruments. Finally, we also hope this study will serve as the basis to synthesize the literature consequences of the COVID-19 pandemic in EMR countries. Consequently, immediate action is required to implement interventions to promote mental wellbeing among HCWs responding to COVID-19.

## Methods

### Study Design

In this study, we used Arksey and O'Malley’s scoping review framework [17], which consists of (1) identifying the research question, (2) identifying relevant studies, (3) study selection, (4) charting the data, and (5) collating, summarizing, and reporting the results.

### Literature Search Strategy

Initially, searches in PubMed were conducted by two researchers (TA and WA) to identify relevant keywords and medical subject headings (MeSH). The search was then performed using six electronic databases (PubMed, CINAHL, Embase, Crossref, Global Health, and PubMed Central) and Google scholar for relevant articles. The search terms were: [“COVID-19,” “SARS-CoV-2,” “Corona virus,” “Corona virus epidemic,” “Corona virus Pandemic,” “COVID-19 pandemic,” “COVID-19 epidemic,” “Psychological impact,” “Anxiety,” “Depression,” “Stress,” “Psychological distress,” “Mental health,” “Eastern Mediterranean Region,” “Middle East,” “Arabs,” “Arabian countries,” “Afghanistan,” “Bahrain,” “Djibouti,” “Egypt,” “Iran (Islamic Republic of),” “Iraq,” “Jordan,” “Kuwait,” “Lebanon,” “Libya,” “Libyan Arab Jamahiriya,” “Morocco,” “Oman,” “Pakistan,” “Healthcare workers,” “HCW”, “Healthcare professionals,” “HCP,” “nurses,” “nurse,” “doctors,” “physicians,” “clinicians,” “medical staff,” “medical workers,” “Healthcare providers,” “providers”].

### Eligibility Criteria

Studies that met the following criteria were included in this review: 1) assessed one or more mental health morbidities such as stress or distress, anxiety, depression, burnout, insomnia, and fear or phobia, 2) cross-sectional design, 3) peer-reviewed article published in the English language between January 2020 and November 2021, 4) all or part of the sample include professional healthcare workers as defined by the WHO [18], and 5) healthcare workers were from the EMR [19], which the WHO defines as 22 nations (Afghanistan, Bahrain, Djibouti, Egypt, Iran, Iraq, Jordan, Kuwait, Lebanon, Libya, Morocco, Oman, Pakistan, Palestine, Qatar, Saudi Arabia, Somalia, Sudan, Syria, Tunisia, United Arab Emirates, Yemen). Conversely, studies that met any of the following criteria were excluded: 1) Comorbid mental health conditions unrelated to the COVID-19 pandemic, 2) Review, interventional, and qualitative studies, 3) HCWs were not part of the population, or could not be separated, 4) HCWs were not from the EMR or 5) Letter to editor, dataset, or preprint articles.

### Identification and Selection of Studies

Two researchers (TA and WA) independently did a literature search using the defined search terms, then compared them to avoid technical errors. Publications were imported into Rayyan [20], and duplicated articles were found and removed before the screening. The screening process was divided into two stages: Title and abstract screening and full-article screening. Three researchers (TA, WA, and CK) independently conducted the screening, and conflicts were resolved by consensus after two researchers (ZN and BM) reviewed it. This process was done three times (March 2021, July 2021, and November 2021).

This study followed the Preferred Reporting Items for Systematic Reviews and Meta-Analyses Extension for Scoping Reviews (PRISMA-ScR) checklist [21]. The checklist can be found in Supplementary Material S1.

### Data Extraction From Included Studies

Following the selection of articles, three researchers (TA, WA, and BM) collected and recorded data in an Excel spreadsheet using a data extraction form. The data extracted from each article were as follows: date of publication, the title of the article, the journal name, the year of publication, the country of data collection, the study period (start and end dates), the study design, the survey form (online, self-administered, or interviewer-administered), the sampling method and power calculation, and the study outcome and instruments used, as well as their validation. The data were double-checked by (ZN), and any identified discrepancies were corrected. Finally, the data were analyzed using descriptive analysis.

## Results

**FIGURE 1:**
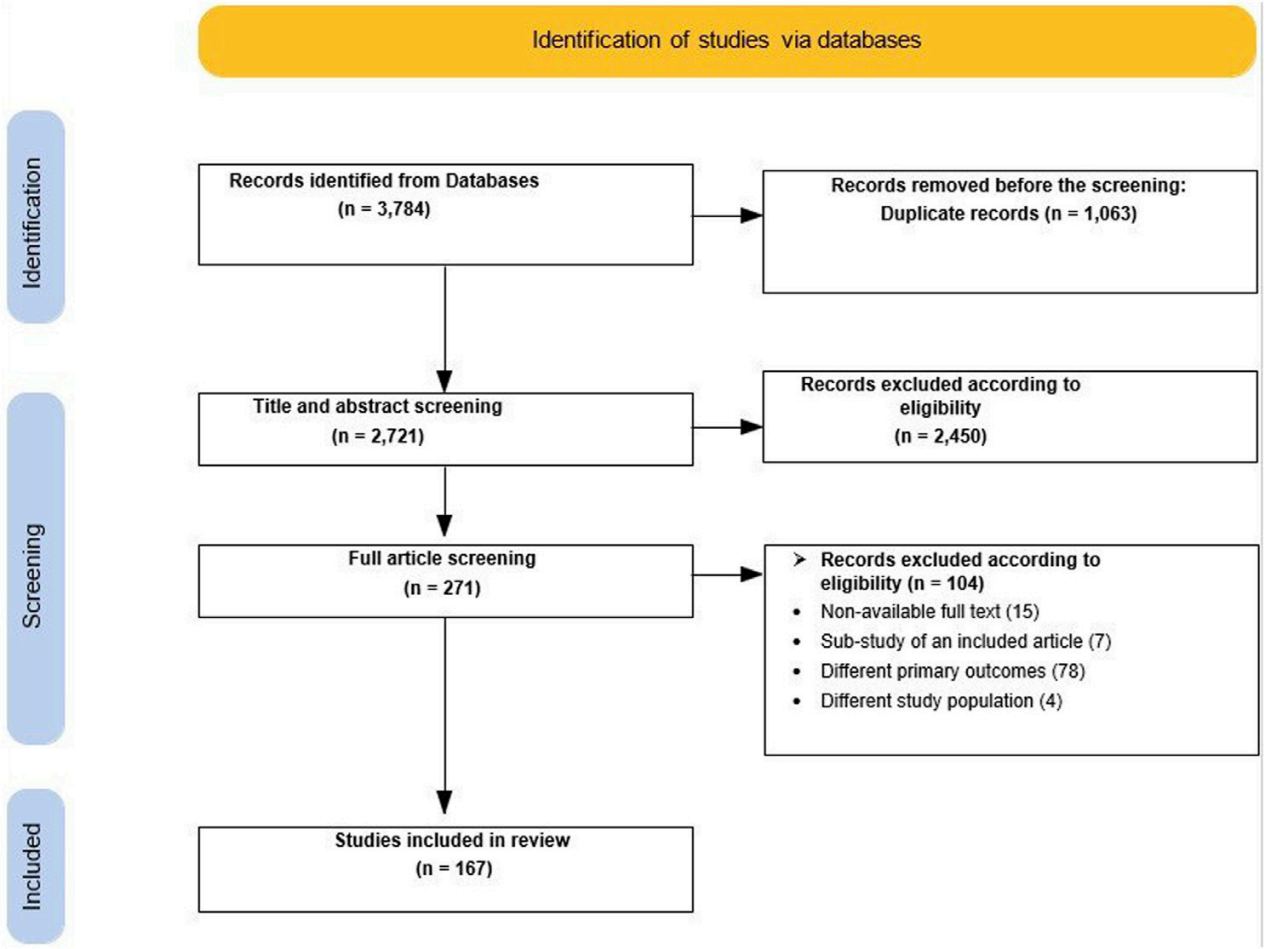
Preferred Reporting Items for Systematic Reviews and Meta-Analyses flow diagram (Palestine, 2022).

Our search yielded 3,784 articles, and after the removal of duplicates, there were 2,721 articles to include in the title abstract screening. One hundred sixty-seven studies were published in the EMR and included in the review. The PRISMA flow diagram in Figure 1 shows the selection of the included articles.

**FIGURE 2:**
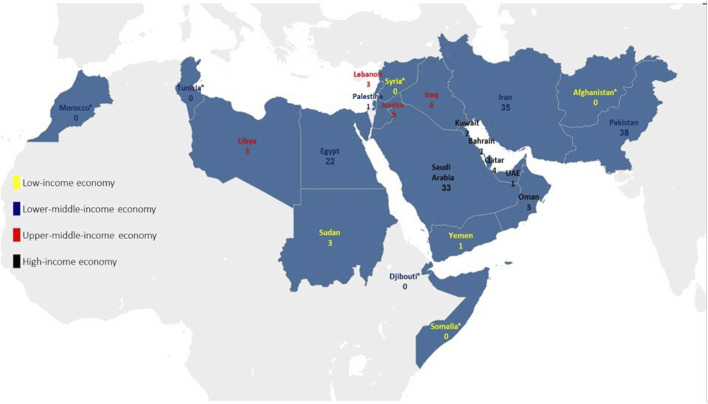
Distribution of publications in the Eastern Mediterranean Region countries. Yellow: Low-income-economy, Blue: Low-middle-income economy, Red: upper-middle-income economy, black: high-income economy (Palestine, 2022).

The countries with an upper middle income (red) and high income (black) reported the most studies since Pakistan and Iran reported 38 and 35 studies, respectively. Saudi Arabia ranks third with 33 studies. The reporting of the studies published in each country in the EMR based on income classification can be seen in Figure 2. Djibouti, Somalia, the Syrian Arab Republic, Tunisia, Morocco, and Afghanistan didn’t conduct any studies considering mental health issues in HCWs. The World Bank Income level classifications show these countries as low-income economies [22].

This review included 94 studies that assessed stress or distress, 93 studies that assessed anxiety and 66 studies that assessed depression; nearly half of all included studies (82 studies) assessed multiple outcomes in the same study. Only a few countries researched burnout, insomnia or sleep quality, and fear or phobia. The distribution of studies on mental health disorders by outcomes and countries is shown in Table 1.

**TABLE 1:** Distribution of studies on mental health disorders by outcomes and countries in the East Mediterranean Region [*n* = 167 studies] (Palestine, 2022).

Outcome Country	Stress/Distress	Anxiety	Depression	Burnout	Insomnia/Sleep quality	Fear/Phobia
Pakistan	19	25	16	3	2	6
Iran	20	20	10	8	2	2
Saudi Arabia	16	12	11	5	3	2
Egypt	13	12	10	4	2	2
Jordan	5	4	4	4	1	0
Oman	3	3	3	0	3	0
Qatar	5	2	2	0	1	0
Sudan	3	3	2	0	0	0
Libya	0	2	2	2	0	0
Lebanon	2	2	1	0	1	0
Iraq	2	1	0	1	1	0
United Arab Emirates	1	1	1	0	1	0
Kuwait	0	1	1	0	1	0
Bahrain	1	0	0	0	1	0
Palestine	1	0	0	0	0	0
Yemen	0	1	0	0	0	0
Multiple countries^a^	3	4	3	0	1	0
Total^b^	94	93	66	27	20	12

^a^ The study was conducted in more than one of the countries in the EMR.

^b^ The total number of studies in this row exceeds the total number of studies included in this review because many studies assessed multiple outcomes in the same article.

The most frequent tools used for anxiety in the included studies were the Generalized Anxiety Disorder (GAD-7) (*n* = 35, 37.6%) and the Depression Anxiety Stress Scale-21 (DASS-21) (*n* = 25, 26.9%). In comparison, 9 (9.7%) of them utilized tools designed by the authors. The most widely used instruments for stress/distress disorders were DASS-21 (*n* = 25, 26.6%) and Perceived Stress Scale-10 (PSS)-10 (n = 23, 24.5%), while 11 (11.7%) of the included studies employed tools created by authors. For depression, the DASS-21 was used in 26 (39.4%) of the studies, followed by the Patient Health Questionnaire-9 (PHQ-9) (*n* = 21, 31.8%). Finally, the Maslach Burnout Inventory (MBI) scale for burnout was employed in 21 (77.8%) of the studies, while Insomnia Severity Index (ISI) tool for insomnia was used in 10 (50.0%) studies. It is worth noting that many of the studies used new measurement tools developed by authors or in previous articles, and a large percentage of these studies provided no information about these tools’ validity and validation process. The types and frequency of measurement tools used to assess mental health issues in EMR are summarized in Table 2.

**TABLE 2:** The distribution of the assessment tools used in the included studies to evaluate different outcomes (Palestine, 2022).

Tools by outcome	Frequency^a^	Percentage (%)
Stress/distress [*n* =94]		
Depression, Anxiety, Stress Scale (DASS-21)	25	26.6
Perceived Stress Scale (PSS)-10	23	24.5
Designed by authors or others	11	11.7
Others	35	37.2
Anxiety [*n* =93]		
Generalized Anxiety Disorder (GAD-7)	35	37.6
Depression, Anxiety, Stress Scale (DASS-21)	25	26.9
Designed by authors or others	9	09.7
Others	24	25.8
Depression [*n* =66]		
Depression, Anxiety, Stress Scale (DASS-21)	26	39.4
Patient Health Questionnaire (PHQ)-9	21	31.8
Hospital Anxiety and Depression Scale (HADS)	7	10.6
Others	12	18.2
Burnout [*n* =27]		
Maslach Burnout Inventory (MBI)	21	77.8
Oldenburg Burnout Inventory (OLBI)	3	11.1
Others	3	11.1
Insomnia [*n* =20]		
Insomnia Severity Index (ISI)	10	50.0
Pittsburgh Sleep Quality Index (PSQI)	6	30.0
Others	4	20.0
Fear [*n* =12]		
Fear of COVID‐19 Scale (FCV‐19S)	6	50.0
Designed by authors or others	5	41.7
Workplace Phobia Scale (WPS)	1	08.3
Used validated Tools (total)		
Stress (94)	52	55.3
Anxiety (93)	41	44.1
Depression (66)	32	48.5
Burnout (27)	17	63.0
Insomnia (20)	6	30.0
Fear/Phobia (12)	7	58.3

^a^ The total number of assessment tools for each outcome differs from the total number of studies included in this review because not all outcomes were studied in each article.

## Discussion

This scoping review summarizes 167 cross-sectional studies to shed light on the mental health issues HCWs faced in the EMR during the COVID-19 pandemic. Reviewing the literature revealed that healthcare workers are at a heightened risk of developing mental health problems in light of the pandemic. However, despite ongoing research on mental health issues resulting from COVID infection, certain aspects of the field remain poorly covered [9, 23, 24].

EMR region is a heavily populated region, coupled with weak health systems, including an absence of medical resources and equipment and a lack of effective prevention programs, all of which contribute to increased workload and psychological stress among its HCW. All mentioned factors make the EMR region an ideal area to investigate the mental health burden of COVID-19 in conjunction with various variables and effects.

We noticed that more than three-quarters of the research in this field comes from Iran, Pakistan, Saudi Arabia, and Egypt. It’s important to mention that these countries have one of the highest publication rate in the EMR region, according to the nature index. Pakistan, Egypt, and Iran’s high publication rates could be attributed to their high population density, research funding, and international collaboration [25, 26]. While there were no publications from Afghanistan, Somalia, the Syrian Arab Republic, Tunisia, Morocco, and Djibouti, the former three countries, notably, have the highest COVID-19 case-death rates in the EMR region [27], putting HCWs at an increased risk of mental health burden. Afghanistan [28], Somalia [29], and the Syrian Arab Republic [30] are areas of conflict. In contrast, Tunisia, Morocco, and Djibouti use French as a second national language, which may account for their scarcity of publications.

Numerous countries in the region are facing a humanitarian conflict. For example, a study conducted in Libya discovered that HCWs overstated the risk of depressive symptoms in such areas [31]. Another Palestinian study found that (74.0%) of HCWs were stressed during the outbreak [32], emphasizing the importance of psychological care and attention.

Outbreaks tend to exacerbate psychological symptoms [33], and being on the frontline puts healthcare workers under additional stress. Perhaps due to feeling unprotected from the virus or its effect on their life and sleep quality [34]. With the emergence of the COVID-19 pandemic, rapid reviews were conducted to draw attention to the psychology of healthcare workers [35, 36]. Our study included the following mental health burdens: anxiety, depression, stress, burnout, fear, and insomnia. Similarly to a review by Kirsten M. Fiest et al., we discovered anxiety is the most studied psychological effect in the COVID-19 outbreak [33]. On the other hand, fear, phobia, and insomnia were among the least investigated outcomes in our pool of studies. For example, in a study done to assess the impact of epidemics, insomnia was identified as the second most prevalent burden behind depression [35]. However, insomnia was under-researched in EMR in this review. As a result, we encourage future research in the EMR region on this subject.

Given the prevalence of cross-sectional studies in research output, it is vital to adopt a uniform methodology to provide reliable information. As a result, many checklists were developed to assess the quality of cross-sectional research [37]. The DASS-21 was the most method of assessing the major mental health issues studied. Because it is simple, quick, and can measure stress, depression, and anxiety all at once, it is more appealing than other, more specific tests [38]. Reliability DASS-21 was good in an Arabic sample [39]. However, Arabic DASS-21 version validation reported it as probabilistic rather than a deterministic measure of distress [40].

This review discovered that only half of the studied outcomes were assessed using validated tools. Tool validation ensures that we measure what is intended to be measured, which varies by tool and population [41]. For instance, we cannot guarantee that the same questionnaire will work similarly for two distinct populations, even if they share a common language. This is due to the two countries’ different cultures. As a result, tool validation and contextualization are critical [42]. When it comes to the mental health burden, there are numerous assessment tools for each illness; this adversity may result in some variances in prevalence and assessing the results difficult [43]. As a result, using non-validated measures adds additional limitations to the process.

Despite the abundance of assessment tools available in the literature, a self-designed questionnaire was used in 10.7% of studies. Developing new tools imposes additional burdens on the authors [44], and given the ubiquity of evaluation tools, one can wonder why authors continue to build their instruments. A variety of factors could account for this finding: a shortage of tools written in the country’s primary language, the absence of validated mental health assessment tools suitable for use in the region, or because the authors are unfamiliar with previous assessment tools or perceive a flaw, we encourage comparative research and a detailed analysis of current assessment tools.

The absence of data can be attributed to either data collection or reporting, resulting in low-quality publications. This constraint is intensified in studies incorporating many articles such as systematic reviews, meta-analyses, and scoping reviews.

### Strengths and Limitations

This study is one of the few that studied the mental health issues in all countries in the EMR. As a result, many articles shed new light on the subject. Furthermore, the study spans 23 months, making it more comprehensive than earlier pandemic research. In addition, it is the only study that incorporates the methodology of articles discussing the mental health issues of HCWs during the COVID-19 pandemic. Despite this, our review is not without limitations because it included only cross-sectional studies, which indicates that we may have missed data obtained through an alternate study design. Furthermore, we included exclusively published publications in English while noting that some nations in the EMR region published studies in other languages, such as French.

### Conclusion and Recommendations

In conclusion, numerous studies have been published examining the impact of the pandemic on healthcare professionals, notably in the EMR region, emphasizing the critical nature of developing strategies to address this issue. However, many of these studies had incomplete data. Therefore, both writers and journals share responsibility for the publication’s quality.

Given the preceding, we recommend enhancing the quality of published work and bridging knowledge gaps, considering that most developing countries have a low publication rate. Countries with the lowest publication rates have the highest demand for evidence of mental health burden. Therefore, we advocate for increased study in these countries, either through research enhancement or by making publishers more supportive of research publications, possibly by waiving publication fees. Furthermore, certain areas, such as insomnia, fear, and phobia, are under-researched during the pandemic, and as a result, we promote additional research on these subjects in particular. Secondly, we advise authors to use checklists while performing cross-sectional studies to ensure correct reporting. In addition, we recommend evaluating the quality of studies included in future systematic reviews. Finally, priority should be given to healthcare workers’ mental health, and governments should continue to invest in measures that aid them in overcoming crises; otherwise, this will threaten the entire healthcare system.
